# Characterization of hypoxia-related molecular clusters and prognostic riskScore for glioma

**DOI:** 10.3389/fonc.2025.1605949

**Published:** 2025-09-22

**Authors:** Xiang Fang, Xinhao Wu, Chengran Xu

**Affiliations:** ^1^ Department of Neurosurgery, Central Hospital Affiliated to Shandong First Medical University, Jinan, Shandong, China; ^2^ Department of Neurosurgery, The First Affiliated Hospital of China Medical University, Shenyang, Liaoning, China

**Keywords:** glioma, riskScore, hypoxia, brain, LIF

## Abstract

**Background:**

Gliomas represent a significant burden in the realm of central nervous system (CNS) malignancies, accounting for approximately 30% of all primary brain tumors and a striking 80% of malignant cases. The incidence of gliomas is observed to escalate with advancing age, exhibiting a marginally higher prevalence in the male population. Among these tumors, high-grade gliomas, particularly glioblastoma multiforme (GBM), are characterized by their aggressive nature and dire prognosis. Conventional therapeutic approaches, including surgical intervention, radiotherapy, and chemotherapy, have demonstrated limited efficacy, underscoring an urgent need for the development of targeted therapies and enhanced mechanistic understanding to improve patient outcomes.

**Methods:**

In this study, we aimed to deepen our understanding of the role of hypoxia, a critical factor in cancer progression, within gliomas. Using comprehensive datasets from The Cancer Genome Atlas (TCGA) and Chinese Glioma Genome Atlas (CGGA), we classified gliomas into two distinct subgroups based on hypoxia-related gene expression profiles: C1 and C2. This classification facilitated a comparative analysis of prognostic outcomes and tumor microenvironment characteristics between the two subgroups.

**Results:**

Our findings revealed that patients within the C1 subgroup exhibited significantly poorer prognoses, with an upregulation of genes intricately linked to various tumor progression pathways. Moreover, the immune microenvironment within the C1 subgroup appeared more favorable for tumor survival and growth, coupled with a notable increase in genomic instability compared to the C2 subgroup. A prognostic scoring system developed from key hypoxia-related factors demonstrated substantial predictive value across multiple cohorts.

**Conclusion:**

Ultimately, we identified four core hub genes—SOCS3, CLCF1, PLAUR, and LIF—whose expression was validated under hypoxic conditions via Western blot analysis in glioma cell lines. This study employs bioinformatics to elucidate glioma subtypes, highlighting significant prognostic and functional disparities. The experimental validation of candidate molecules paves the way for future research aimed at unraveling their roles and underlying mechanisms in glioma pathophysiology, potentially guiding novel therapeutic strategies.

## Introduction

Glioma is a common cancer that affects the brain and central nervous system, accounting for approximately 30% of all primary brain tumors and 80% of all malignant brain tumors (1–3). Notably, the incidence of glioma increases with age, with slightly higher rates in males than females (4, 5). Unfortunately, the outcomes for these patients are frequently unfavorable. High-grade gliomas, particularly glioblastomas (GBM), are extremely aggressive and are life-threatening. Treatment options for gliomas include surgery, radiation therapy, chemotherapy, or a combination of these approaches (6, 7). However, the limitations of the current treatments underscore the need for continued research and development of new, more targeted treatment approaches. Therefore, improving treatment strategies and understanding the underlying mechanisms of gliomas are vital.

Hypoxia, or low oxygen tension, is an important factor in the growth, development, and progression of cancer (8–12). Several pathways are involved in the response of cancer cells to hypoxia, including the hypoxia-inducible factor (HIF) pathway, which promotes angiogenesis, cell survival, and tumor invasion (9, 13–15). Furthermore, hypoxia affects the immune response, making tumor cells more resistant to immune cell recognition and attack (16, 17). Therefore, targeting the hypoxic pathways in cancer therapy has emerged as an attractive approach for improving cancer treatment outcomes. Importantly, several types of cancers, such as breast cancer, non-small cell lung cancer, and malignant melanoma, have been reported to be affected by hypoxia (18–24). Nevertheless, advancements are crucial for a deeper comprehension of hypoxia’s impact on gliomas. This encompasses elucidating the intricate hypoxic pathways, precisely quantifying hypoxia’s extent and distribution, and innovating effective therapies targeting hypoxia in gliomas.

By employing unsupervised clustering analysis utilizing hypoxia-related gene expression profiles, we successfully categorized gliomas into two distinct subtypes. Each subtype exhibits unique somatic alterations, immune cell composition, metabolic characteristics, and clinical outcomes. Through co-expression network analysis, we identified four crucial hub genes, three of which were upregulated in response to hypoxia treatment in glioma cell lines. These findings have the potential to greatly advance clinical diagnosis and mechanistic research in the field of glioma.

## Materials and methods

### Patients and samples

TCGA (The Cancer Genome Atlas) is a pan-cancer research program initiated jointly by the NCI and NHGRI in the United States, which includes genomic, transcriptomic, and clinical data from 33 types of cancer and over 11,000 patients. The Chinese Glioma Genome Atlas (CGGA) is a specialized database led by Beijing Tiantan Hospital, focusing on multi-omics research of primary and recurrent brain gliomas. Its core integrates genomic, transcriptomic, epigenetic data, and complete clinical follow-up information, with a sample size exceeding 2,000 cases (as of 2023). Data repositories, including TCGA (LGG and GBM) and CGGA (mRNA_325 and mRNA_301) databases, were searched for available glioma genomics, transcriptomics, and clinical information. Our research scope includes patients from WHO stage II to IV. The aforementioned datasets were downloaded from various websites (https://xenabrowser.net/datapages/ and http://www.cgga.org.cn/).

### Identification of hypoxia cluster

The WINTER_HYPOXIA_METAGENE gene set obtained from the Molecular Signatures Database (MSigDB) (https://www.gsea-msigdb.org/gsea/msigdb/index.jsp) was selected for k-means clustering. Normalized expression data were subjected to k-means clustering using the Consensus ClusterPlus R package. The optimal number of clusters was determined by selecting the k-value at which the magnitude of the cophenetic correlation coefficient decreased. Additionally, principal component analysis (PCA) was employed to gain a better understanding of variations between clusters.

### Differentially expressed gene analysis and functional enrichment

Differential expression analyses were performed using the “DESeq2” package. In this analysis, genes with an absolute log2 fold change greater than 1 and a false discovery rate (FDR) <0.05 were considered as differentially expressed genes between two clusters. Gene ontology and Kyoto Encyclopedia of Genes and Genomes (KEGG) pathway analysis was conducted using the “ClusterProfiler” R package, with a cutoff value of FDR < 0.05 (25). To investigate the difference in biological process terms between two clusters, GSEA and GSVA were performed using the R package, considering the gene sets of “h.all.v7.5. symbols” and “c2.cp.kegg.v7.5. symbols” downloaded from MSigDB.

### Estimation of immune infiltration and tumor purity

The CIBERSORT algorithm was used to estimate the relative fractions of the 22 immune cell types in each sample (26). ESTIMATE was used to evaluate the immune cells and stromal contents of each sample (27).

### Somatic mutation identification

Somatic mutation data of all patients categorized under the “Masked Somatic Mutation” category in TCGA were processed using VARSCAN software (https://portal.gdc.cancer.gov/). Mutation analysis and visualization were performed using the “maftools” package. To detect differences in copy number alterations between the subtypes, GISTIC2.0 analysis was conducted. Here, loci with a GISTIC value greater than 1 or less than -1 were defined as amplification or deletion, respectively.

### Weighted co-expression network

A normalized expression matrix was used to construct a weighted co-expression network (WGCNA) using R package (28). A co-expression network was created using the blockwiseModules function with default parameters. The modules were evaluated for significance based on the correlation between module eigengenes and information using Pearson’s test. Hub genes were then chosen based on the modular connectivity of each gene and its relationship with the phenotypic traits in the hub module.

### Cell lines and culture

LN229 and U118 cells were procured from the American Type Culture Collection (Rockville, MD) and maintained in Dulbecco’s modified Eagle’s medium supplemented with high glucose, sodium pyruvate, 10% fetal bovine serum, and 1% penicillin–streptomycin. To simulate hypoxic conditions, cells were cultured in a hypoxia chamber with 94% N_2_, 5% CO_2_, and 1% O_2_ at 37°C.

### Western blotting

Protein extraction and western blot analysis were performed as previously described (29). The antibodies used were anti-HIF-1 (1:5000, 20960-1-AP), anti-SOCS3 (1:2000, 14025-1-AP), anti-PLAUR (1:1000, 10286-1-AP), and anti-LIF (1:500, 26757-1-AP), all of which were obtained from Proteintech Group (Wuhan Sanying, China). The antibodies were validated using a commercial vendor. The bands on each membrane were detected using an ECL kit from Beyotime Biotechnology (Beijing, China), and ImageJ software (National Institutes of Health, Bethesda, MD, USA) was used for quantification.

### Bioinformatic and statistical analyses

To detect survival differences between clusters, Kaplan–Meier analysis with log-rank tests was performed. Unpaired Student’s t-tests were used for normally distributed variables, and Wilcoxon rank-sum tests were conducted for non-normally distributed variables when comparing the two groups. The Benjamini–Hochberg method was used to adjust the P-value. For the univariate analysis, we selected factors known to affect outcomes and patient characteristics. Cox regression analysis was then conducted with age, sex, and grade as factors. Cox proportional hazard models were used to examine the impact of various risk factors on event outcomes, and the reliability of the model was assessed using the Schoenfeld residual. To adjust for explanatory confounding variables that were prognostic in the univariate analysis, Cox multivariate analysis was conducted. A hypoxia-related risk score was established by including normalized gene expression values weighted by their Least Absolute Shrinkage and Selection Operator (LASSO) Cox coefficients (30). All statistical analyses were performed using R software, and statistical significance was considered at a P-value < 0.05.

## Results

### Consensus clustering reveals two hypoxia clusters in glioma

**Figure 1:**
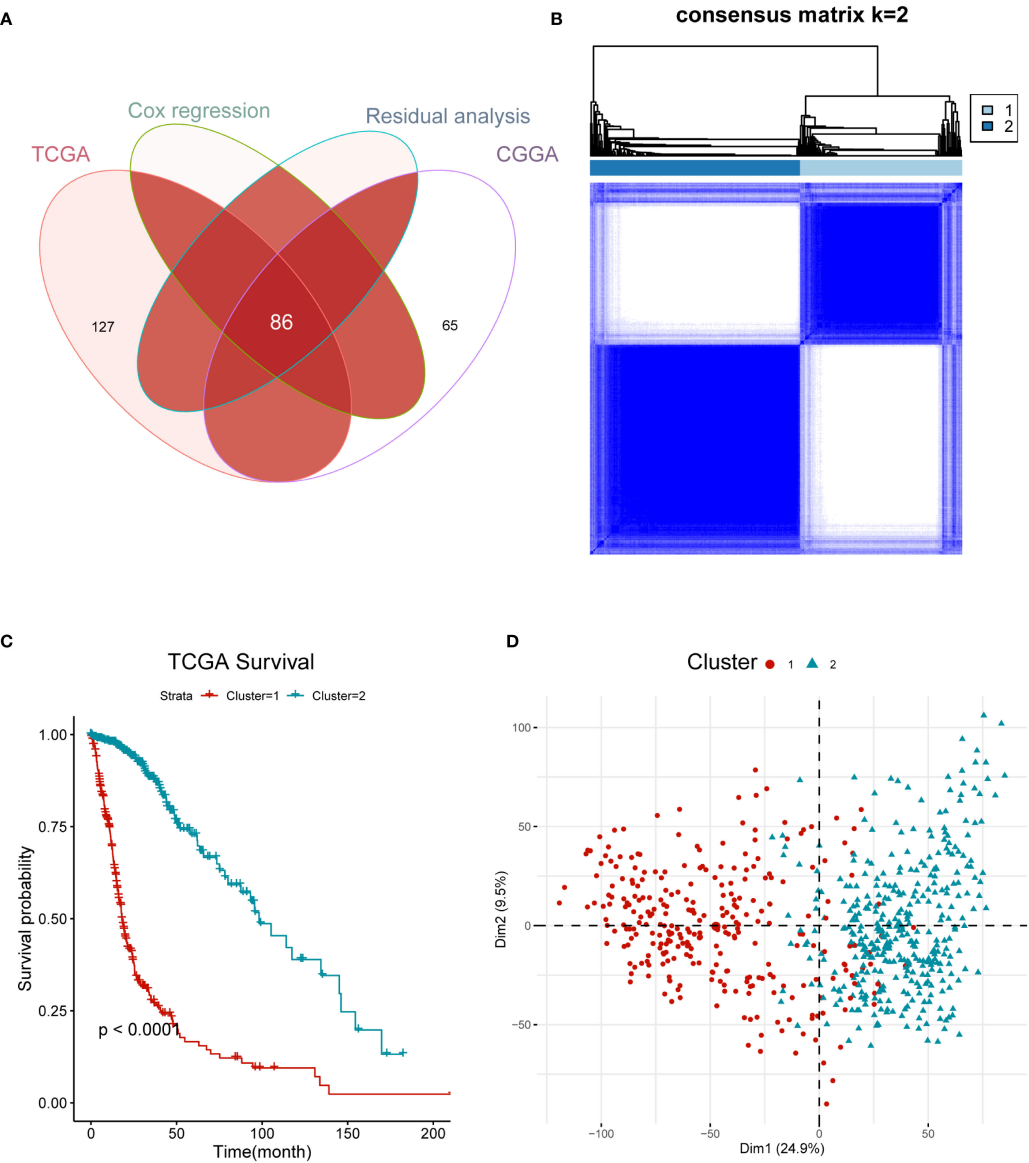
Hypoxia gene profile of glioma yielded two clusters in the TCGA cohort. **(A)** Venn diagram showing 86 candidate gene. **(B)** Heatmap displaying consensus clustering with the robust classification (k = 2). **(C)** Survival analysis of C1 and C2 based on OS. The P-value is calculated by the log-rank test between clusters. **(D)** Principal component analysis (PCA) of two clusters using whole transcriptome data.

To identify the heterogeneity of hypoxia within gliomas, 240 hypoxia-related genes were obtained from MSigDB for clustering analysis. We conducted univariate Cox regression and residual analyses to identify genes with prognostic significance, resulting in a total of 86 candidate genes for subsequent clustering analysis in both TCGA and CGGA cohorts (**Figure 1A**, **Supplementary Table**). Using an unsupervised consensus clustering analysis, we identified two clusters, C1 and C2 (**Figure 1B**). Survival analysis revealed that patients with the C1 subtype were likely to have worse outcomes than those with the C2 subtype (P <0.0001, log-rank test, **Figure 1C**). Furthermore, we performed PCA to confirm the assignment of clusters and robust differences in expression patterns between the two clusters (**Figure 1D**).

### Identification of differentially expressed genes and functional analysis

**Figure 2:**
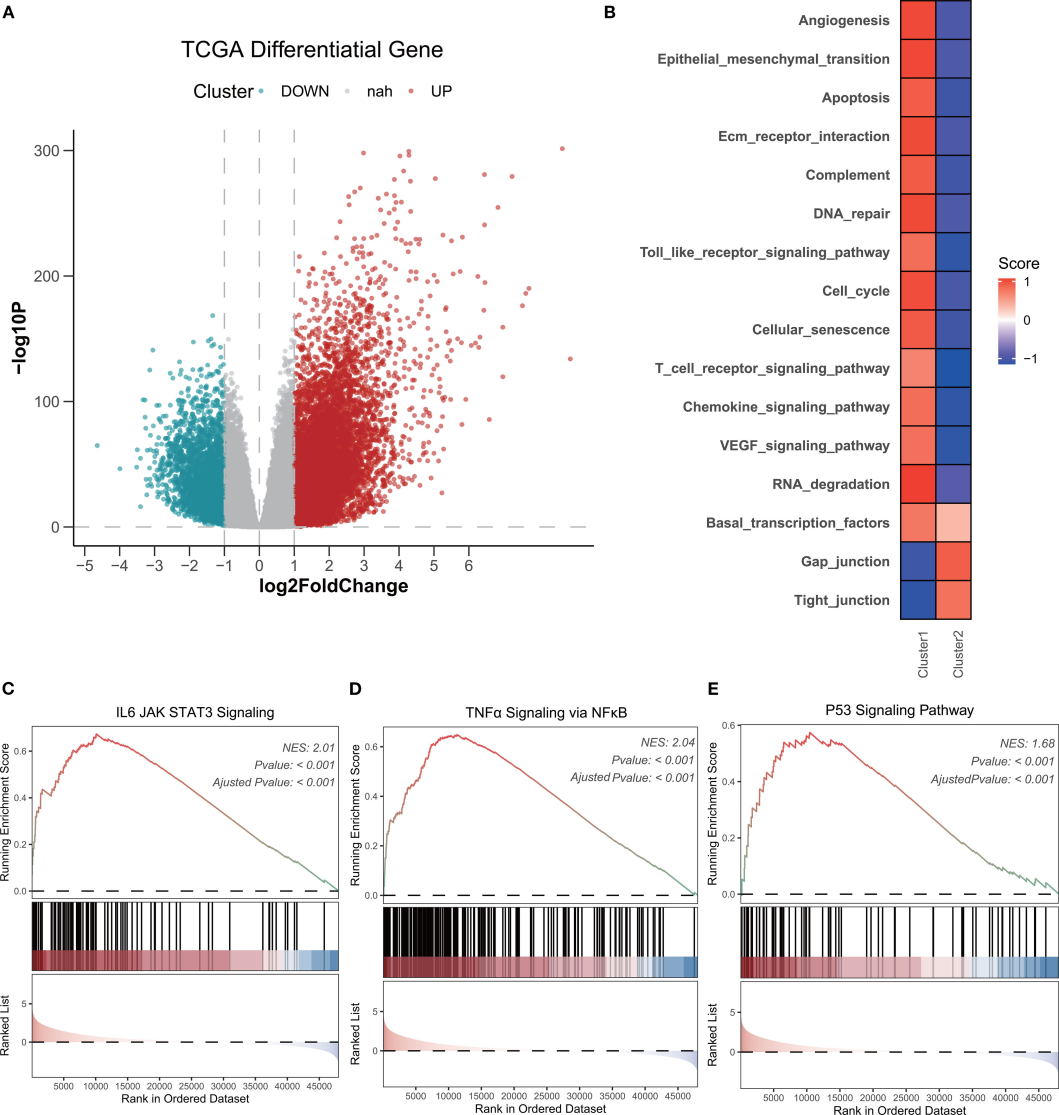
Functional insights of distinct hypoxia clusters in glioma. **(A)** Volcano plots showing genes that are differentially expressed in C1 or C2 samples. Red dots indicate genes upregulated in C1; blue dots indicate genes upregulated in C2. **(B)** Comparison of KEGG functions scores(calculated by GSVA analyze) between C1 and C2 (the color of the squares indicates the high and low average scores of all samples within each cluster). **(C-E)** GSEA analyses of HALLMARK gene set between C1 and C2, including JAK-STAT pathway, NF-κb pathway and P53 signaling.

We performed differential analysis using C1 as the experimental group. Based on the cutoff criteria of |log2 (fold change) | >1.0 and FDR <0.01 using R package “DESeq2”, we identified a total of 10,482 differentially expressed genes (7,622 upregulated and 2,860 downregulated), as shown in the volcano plot (**Figure 2A**). To explore the functional status, we calculated KEGG signaling scores using the GSVA method and ran a GSEA analysis for the HALLMARKER pathways. Our results revealed that tumor malignancy-related signaling pathways, such as angiogenesis, epithelial–mesenchymal transition, and the cell cycle, were enriched in C1. In contrast, gap and tight junctions showed lower enrichment scores (**Figure 2B**). Furthermore, C1 exhibited higher enrichment of the Janus Kinase/Signal Transducer and Activator of Transcription (JAK-STAT), Tumor Necrosis Factor alpha/Nuclear Factor kappa-light-chain-enhancer of activated B cells (NF-κB), and P53 pathways (**Figures 2C-E**). In summary, our findings suggest that C1 glioma cells exhibit a higher degree of hypoxia and a more significant malignant phenotype.

### Immune characteristics and hypoxia clusters

**Figure 3:**
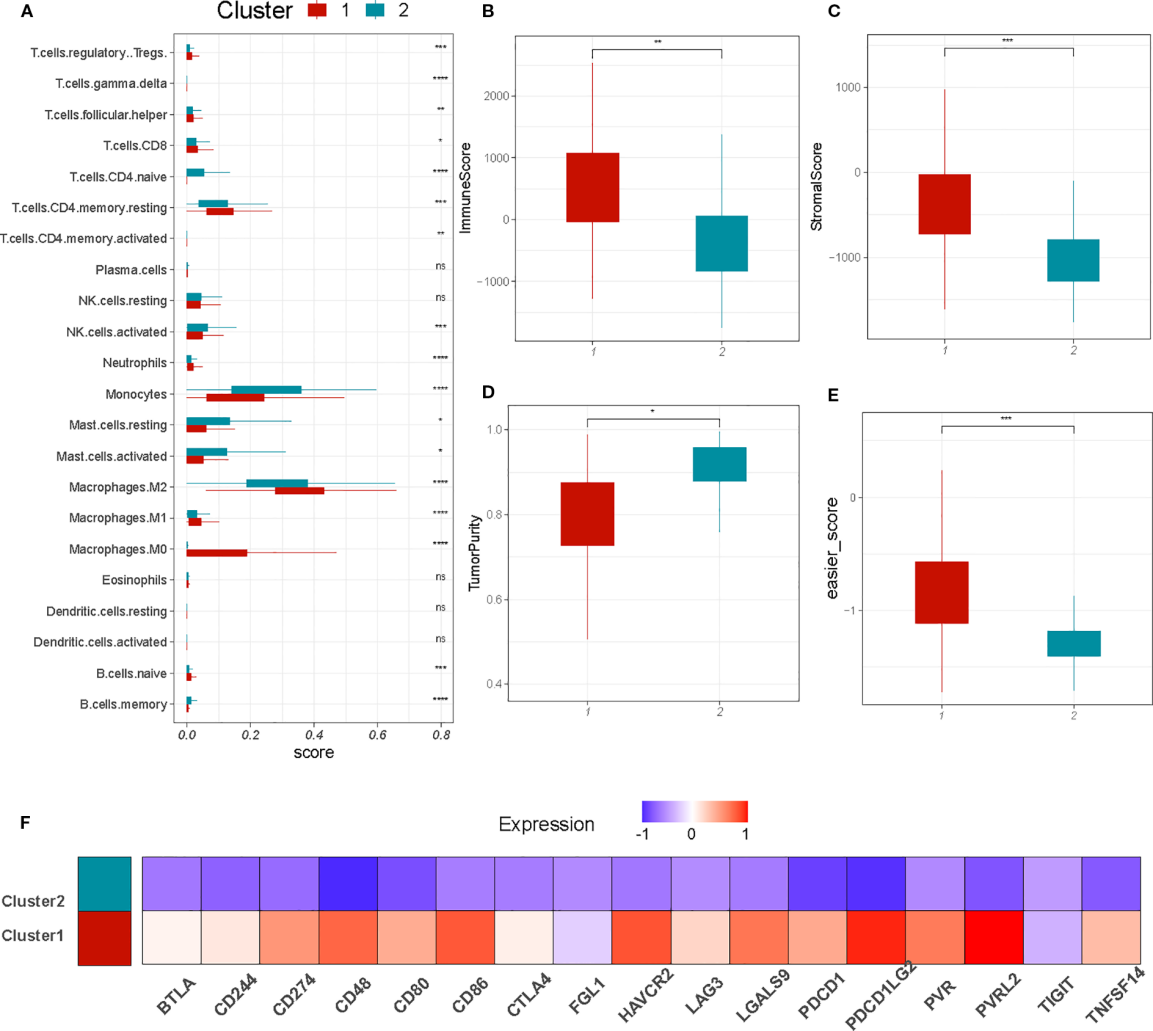
Investigation of the immunologic intertumoral heterogeneity. **(A)** Relative proportion of 22 infiltrating immune cells estimated by CIBERSORT algorism between C1 and C2. **(B-D)**. Comparison of immune, stromal, and tumor purity scores from ESTIMATE for C1 and C2. **(E)** Immune therapy response scores(calculated by EaSIeR algorism) between C1 and C2. **(F)** Heatmap displaying the average expression levels of inhibitive checkpoint genes. *P < 0.05; **P < 0.01;***P < 0.001.

Next, we utilized several previously reported immune-related tools to decode the immune infiltration of the immune subtypes. First, we used the CIBERSORT method to compare the composition of infiltrating immune cells between the groups. C2 had a higher percentage of monocytes and plasma cells compared to C1, whereas C1 had a higher percentage of macrophages, including M0,M1 and M2(**Figure 3A**). On the other hand, the ratio of CD4+ T cells and CD8+ T cells in C1 is slightly higher than that in C2, although the total amount is relatively low. Subsequently, we determined stromal and immune scores using the ESTIMATE algorithm. The C1 cluster showed higher immune and stromal scores and lower tumor purity than did cluster C2 (**Figures 3B-D**). Furthermore, we use the EaSIeR package, a tool based on system tumor microenvironment characteristics, to quantify immune cell composition and predict different features of immune response through intracellular and intercellular communication,We discovered that the C1 cluster might have a higher immunotherapy response score compared to cluster C2 (**Figure 3E**) (31). In addition, most classical checkpoint genes, such as *PDL1*, *CTLA4*, *CD86*, *HAVCR2*, *LGALS9*, and *CD48*, were highly expressed in C1 tumors (**Figure 3F**), indicating an increased level of immunosuppression in these tumors.

### Genomic alterations of hypoxia clusters

**Figure 4:**
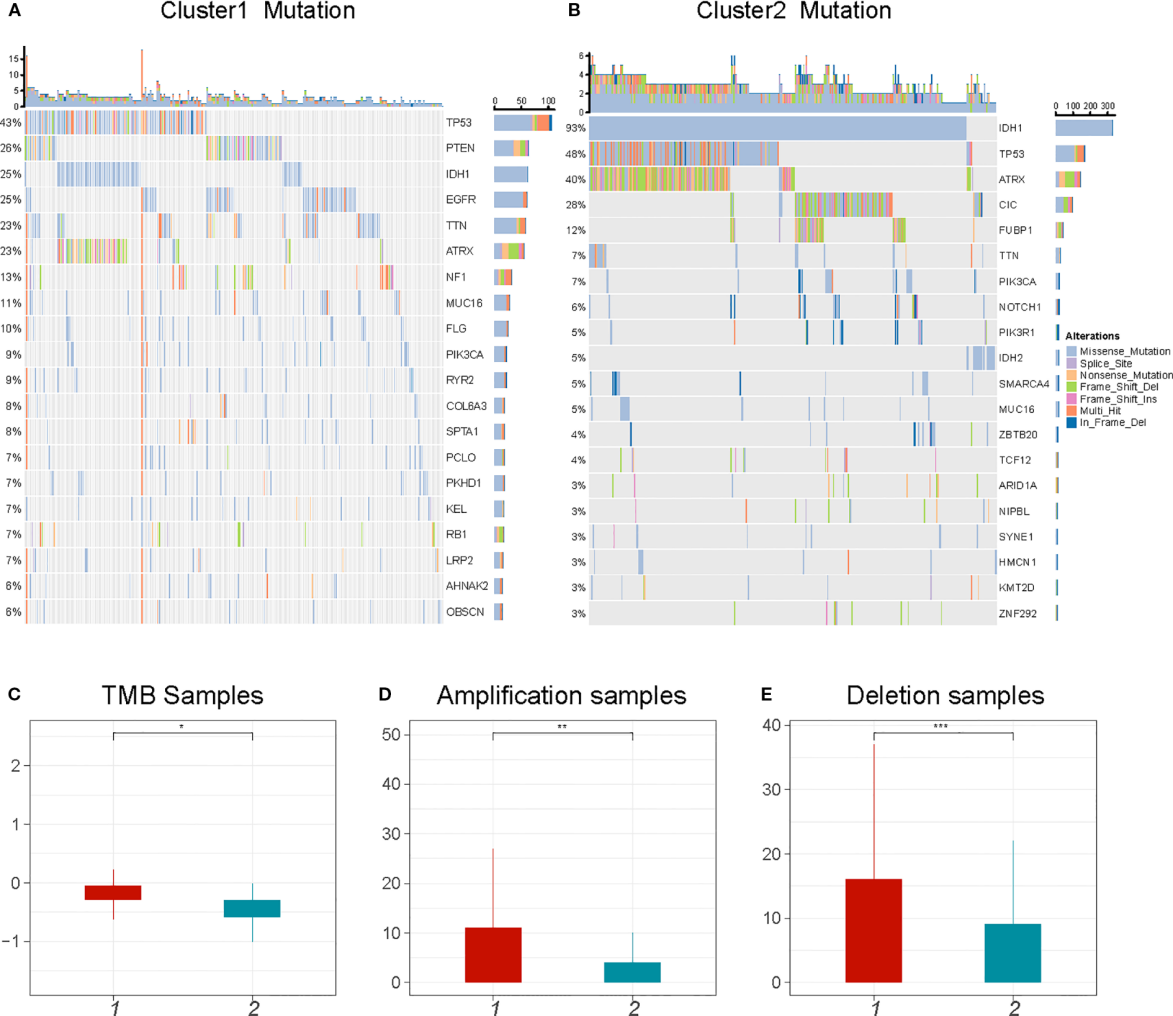
Comparison of genomic alterations between C1 and C2. **(A, B)** The top 20 mutated genes in glioma between C1 and C2. The colors of rectangles in the body of the heatmap indicate different types of somatic mutations. **(C-E)** The number of mutations and copy number aberrations including tumor mutation burdens, amplifications and deletions *P < 0.05; **P < 0.01;***P < 0.001.

It has been reported that tumor genomic mutations correlate with oxygen supply levels (32, 33). We analyzed the differences in gene mutations between the two clusters and specifically looked at the top 20 genes with different mutation frequencies in gliomas. C2 showed significantly higher frequencies of *IDH1* and *ATRX* (93% and 40%, respectively) than did C1 (25% and 23%, respectively). The mutation rate of *TP53* in C1 was similar to that in C2 (43%–48%) (**Figures 4A, B**). In addition, there was significant heterogeneity in the CNV profiles between the two clusters. C1 had more amplified and deleted variant samples as well as a higher tumor mutational burden (TMB) than did C2 (**Figures 4C-E**). These results indicate that gene mutations may be associated with the hypoxic cluster phenotype.

### Construction of the prognostic model

**Figure 5:**
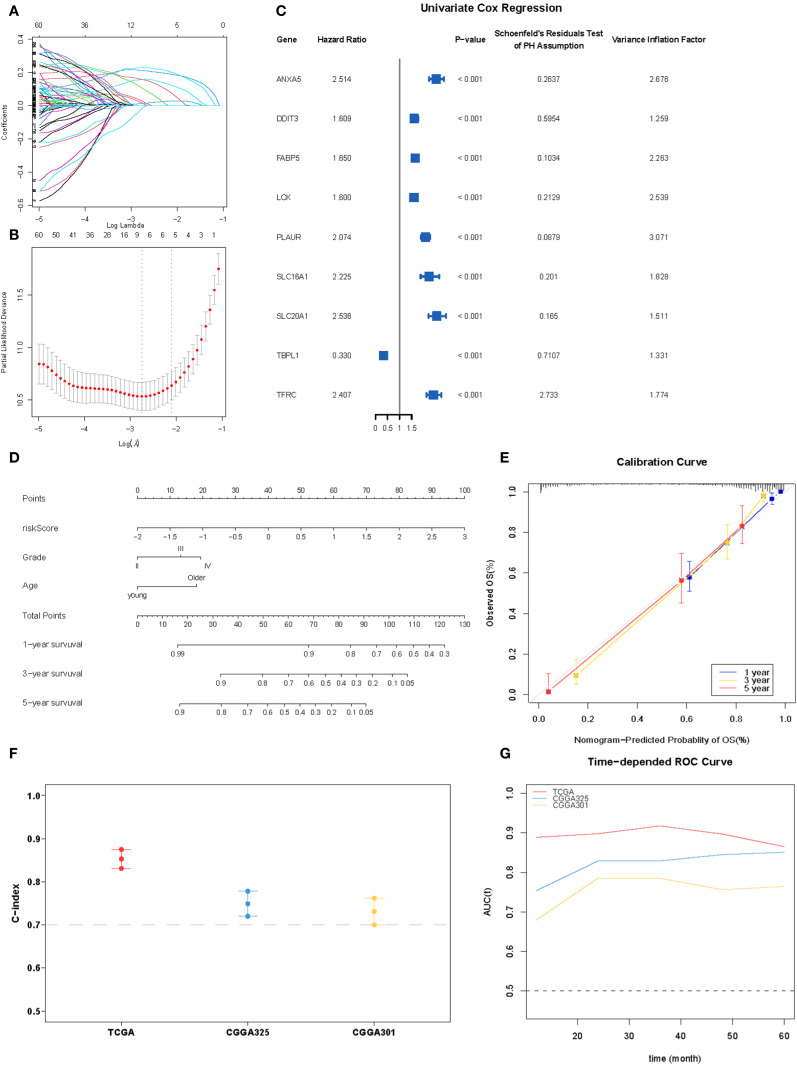
Construction of the hypoxia-associated prognostic model. **(A, B)** Partial likelihood deviance for the LASSO regression and Lasso regression analysis. **(C)** Forest plot for nine robust candidate genes. **(D)**. Nomogram obtained using multivariate Cox regression analysis for predicting the proportion of patients with OS. **(E)** Plots depicting the calibration of the model in terms of the agreement between the predicted and observed OS. The model performance is shown in the plot relative to the 45-degree line, which represents a perfect prediction. **(F)** Concordance indices (C-index) of the nomogram-based signature in TCGA, CGGA_325, and CGGA301 datasets. **(G)** ROC curve AUC plotted for different durations of OS for nomogram-based signatures in TCGA, CGGA_325, and CGGA301 datasets.

To refine prognostic biomarkers from the initial 86 hypoxia-related genes, we employed a multi-step filtering approach. First, genes were subjected to univariate Cox proportional hazards regression (P < 0.05), followed by multivariate Cox regression and Schoenfeld’s residuals test (P > 0.05 for proportionality assumption), yielding 60 candidate genes. Subsequently, a Least Absolute Shrinkage and Selection Operator (LASSO) Cox proportional hazards regression with 10-fold cross-validation was applied to this candidate set to mitigate overfitting and identify core prognostic features. The optimal penalization coefficient lambda (lambda.min= –2.7894) minimized the cross-validation error, resulting in a 9-gene prognostic signature (*ANXA5*, *DDIT3*, *FABP5*, *LOX*, *PLAUR*, *SLC16A1*, *SLC20A1*, *TBPL1*, and *TFRC*). Their prognostic significance was statistically meaningful and consistent with the PH assumption, and they were also free from multicollinearity (**Figures 5A–C**). A prognostic index was calculated for all cancer samples using the following formula: risk score = ANXA5×0.15648040+DDIT3×0.01273471+FABP5×0.21023267+LOX×0.0112542+PLAUR×0.22763644+SLC16A1×0.02450256+SLC20A1×0.10289532+TBPL1×-0.01552882+TFRC×0.1555086.

In the multivariate Cox proportional hazards analysis (**Table 1**), the riskScore was identified as an independent prognostic biomarker (HR = 1.92, 95% CI = 1.69–2.19, p <0.001). To improve prognostic prediction in patients with glioma, we developed a nomogram by integrating three independent predictors – risk score, age, and grade – into a multivariate Cox regression model. We evaluated and validated this nomogram using data from TCGA, CGGA_325, and CGGA_301 databases. The nomogram generated a score that predicted the 1-, 3-, and 5-year overall survival (OS) rates for individual patients (**Figure 5D**). The performance of the nomogram in predicting patient OS was evaluated using a calibration plot, which demonstrated that it accurately predicted patient survival according to an ideal model (**Figure 5E**). The concordance indices for predicting OS with the nomogram model were 0.857, 0.775, and 0.724 in TCGA, CGGA_325, and CGGA_301 cohorts, respectively (**Figure 5F**), indicating good predictive accuracy. Furthermore, for the 1- to 5-year OS prediction, the time-dependent receiver operating characteristic curve demonstrated excellent predictive performance (area under the curve >0.7) across all three cohorts (**Figure 5G**).

**Table 1:** Univariate and multivariate Cox regression analyses of riskScore with WHO grade, age, and sex.

Variables	Univariable cox				Multivariable cox			
	Hazard ratio	Lower .95% CI	Upper .95% CI	P. value	Hazard ratio	Lower .95% CI	Upper .95% CI	P. value
riskScore	1.92	1.69	2.19	p<0.001	1.39	1.19	1.63	p<0.001
Age ≤ 42 *vs* > 43	0.52	0.40	0.68	p<0.001	0.96	0.72	1.28	p=0.776
Grade II *vs* III	3.65	2.38	5.60	p<0.001	3.18	2.06	4.92	p<0.001
Grade IV *vs* III	8.97	6.03	13.36	p<0.001	6.17	3.96	9.61	p<0.001
Gender	0.93	0.71	1.22	p=0.602				

### Landscape of riskScore associations: clinical subgroups, functional activities, and metabolic signatures

**Figure 6:**
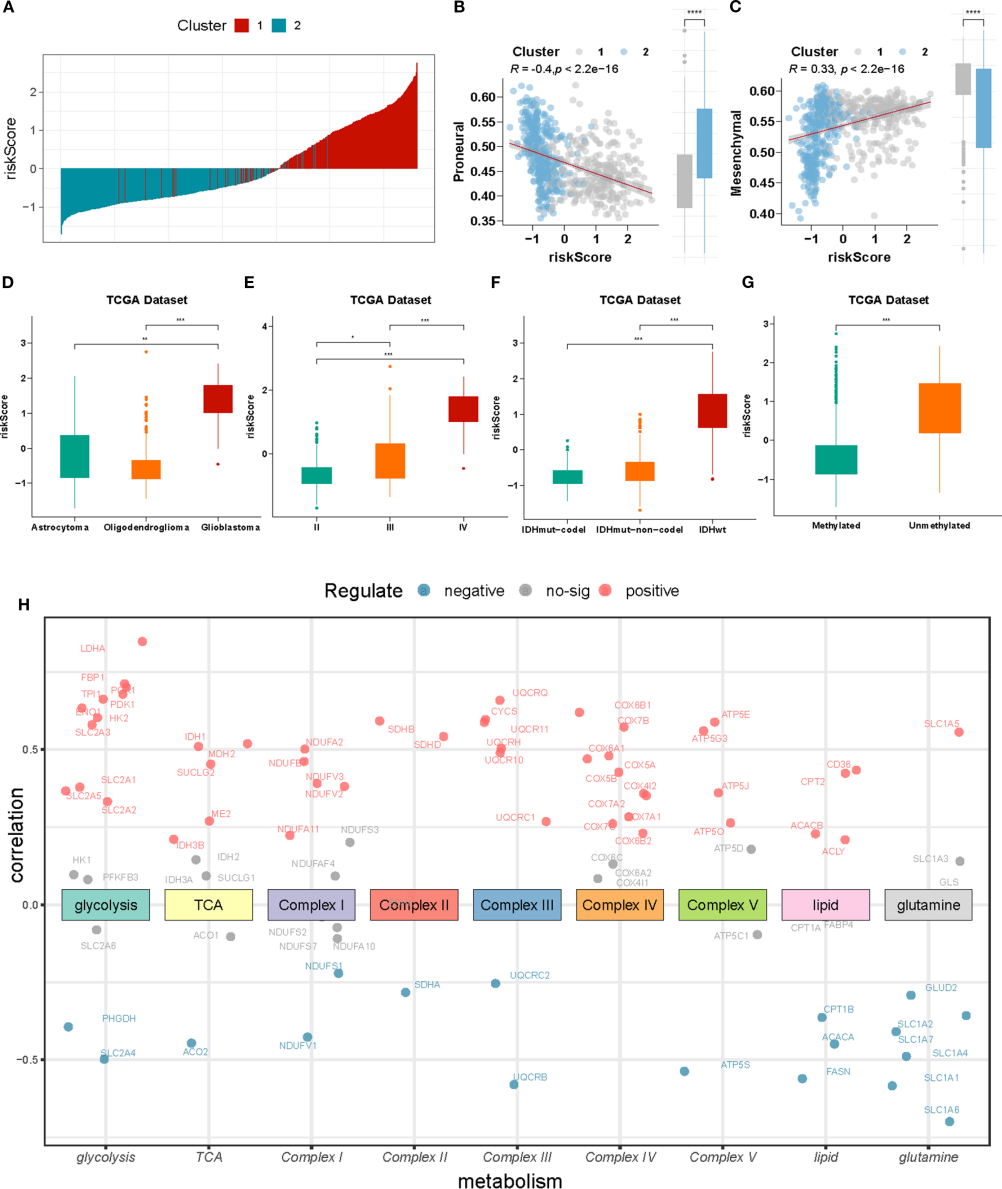
Relationship between clinical features of gliomas and nomogram-based signature. **(A)** Distribution of riskScore and hypoxia-related clusters. **(B, C)** The correlation between riskScore and proneural and mesenchymal scores. **(D-G)** Boxplot indicating riskScore in different histological type, WHO grades, IDH-1 with 1p19q status, and MGMT promoter methylation from TCGA dataset. **(H)** Correlations of the riskScore with different metabolism marker gene expression, the color of the scatter points represents the significance of the Pearson correlation analysis, with red indicating a positive correlation, blue indicating a negative correlation, and gray indicating no correlation. *P < 0.05; **P < 0.01;***P < 0.001.

Next, we analyzed the relationship between risk score and clinical parameters. As shown in **Figure 6A**, the risk score of the majority of the C1 cluster was higher than that of the C2 cluster. Additionally, there was a negative correlation between the risk score and the Proneural score of glioblastoma (r = -0.4, p <0.001), whereas a positive correlation was observed with the mesenchymal score (r = 0.33, p <0.001) **Figures 6B, C**. Moreover, the risk score was higher in patients with GBM, World Health Organization grade IV, wild-type IDH-1, and non-methylated MGMT promoter **Figures 6D–G**.

Hypoxia can also alter the expression of genes involved in energy metabolism, leading to changes in protein and enzyme levels. To identify differences in energy metabolism, we examined seven central metabolic pathways: glycolysis, tricarboxylic acid cycle, oxidative phosphorylation, lipid, and glutaminolysis. As shown in **Figure 6H**, most markers of glycolysis and oxidative phosphorylation were positively correlated with the risk score (r >0.2, P <0.001). However, further studies are required to investigate the global effects of hypoxia on metabolism.

### Identification of hub genes through WGCNA analysis

**Figure 7:**
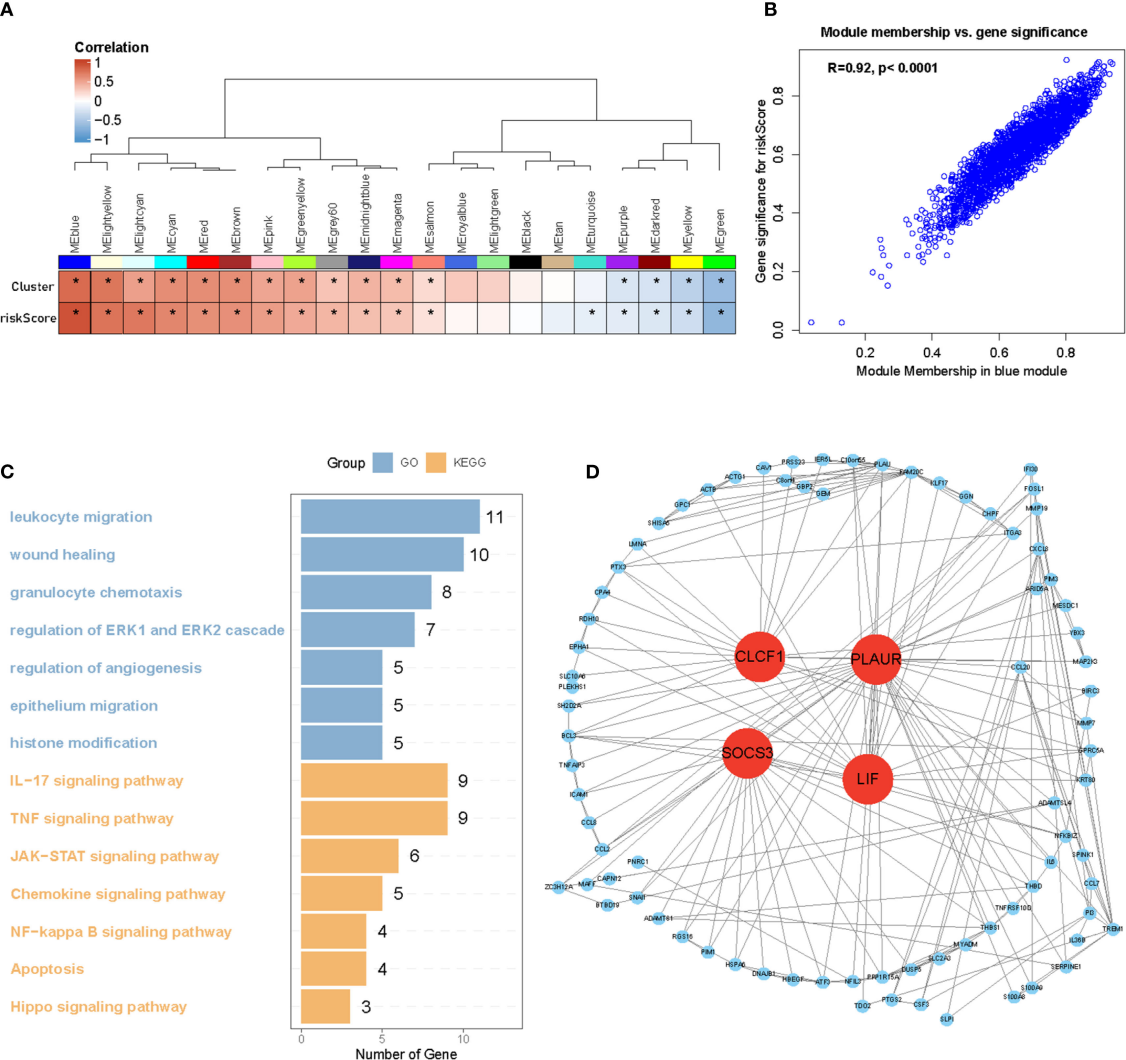
Identification of four hub genes of Hypoxia cluster. **(A, B)** WGCNA analysis identified the blue module as the highest correlated module with hypoxia (r = 0.92, p <0.001). **(C)** GO and KEGG analysis of blue module genes. **(D)** Hub genes of blue modules filtered by degree of connectivity. *P < 0.05.

**Figure 8:**
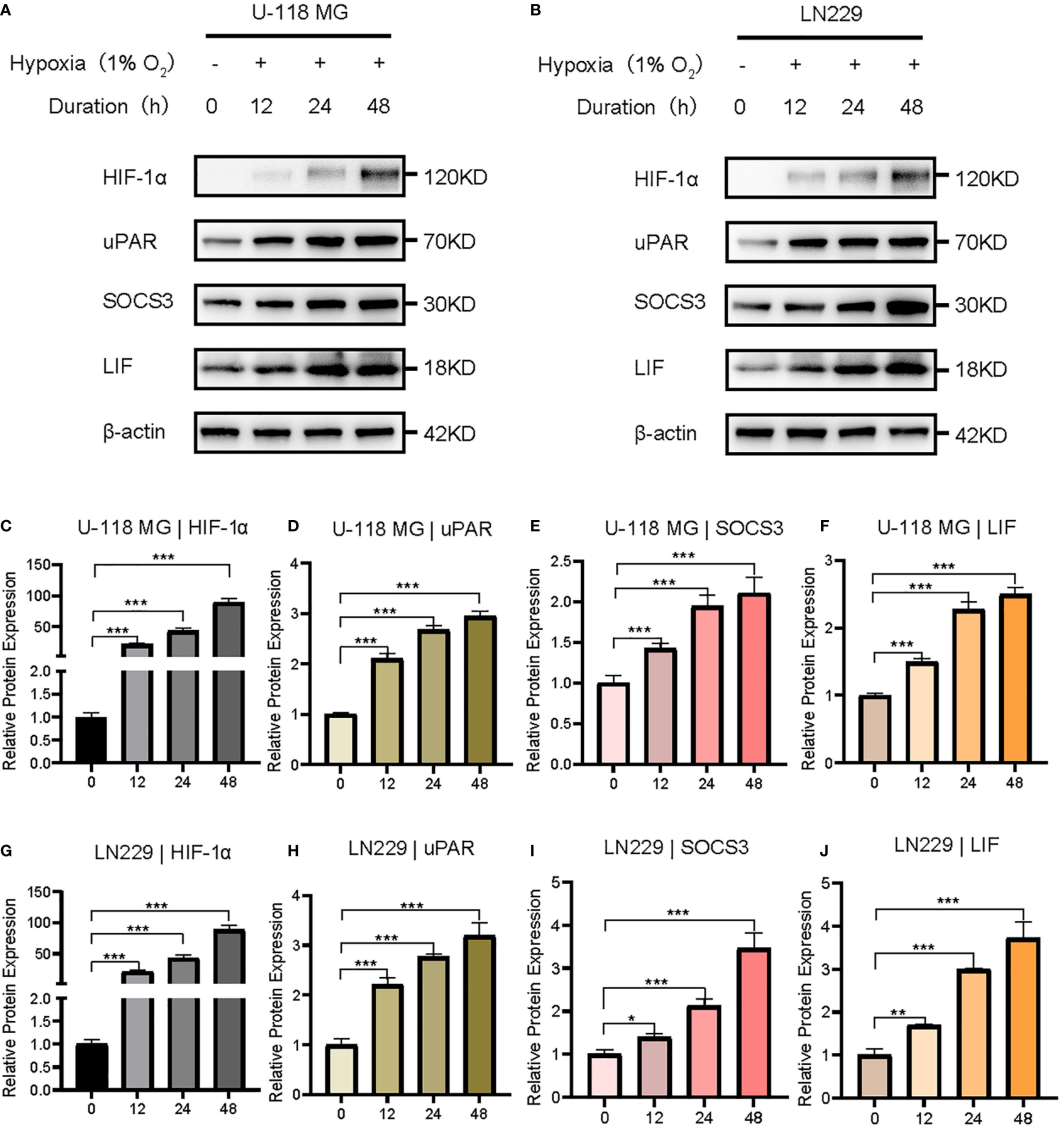
Western blotting validation of HIF-1 and hub genes in glioma cell lines under hypoxic conditions. **(A, B)** Western blotting showing that the protein expressions of HIF-1, PLAUR, SOCS3, and LIF in U118MGand LN229 celllines are upregulated under hypoxic conditions. **(C-J)** Using Image J to perform semi-quantification of the PLAUR, SOCS3, and LIF protein bands in **Figure 8**, calculating the relative expression of each protein in the cells based on the quantitative values. All data were analyzed using the t-test and were shown as the mean ± SD (two independent experiments). *P < 0.05; **P < 0.01;***P < 0.001.

Weighted gene co-expression network analysis (WGCNA) was performed to identify hypoxia-regulated hub genes using transcriptomic data from the TCGA samples (n=661 samples). After quality control and normalization, 19,583 protein-coding genes were retained for network construction. A soft thresholding power of β=10 was selected to achieve scale-free topology (scale-free fit index >0.8);. Hierarchical clustering with dynamic tree cutting (minModuleSize = 50) identified 22 distinct co-expression modules. Among these modules, the blue one was found to have the highest correlation with hypoxia (r = 0.92, p <0.0001) (**Figures 7A, B**). Biological process analysis revealed that genes in the blue module were enriched in wound healing, angiogenesis regulation, and histone modification. Furthermore, KEGG pathway analysis demonstrated that the genes were associated with the JAK-STAT and NF-κB signaling pathways (**Figure 7C**). Finally, after identifying hub genes with a high degree of connectivity in the blue module, we found that *CLCF1*, *PLAUR*, *SOCS3*, and *LIF* were the top four hub genes (**Figure 7D**). HIF-1 is the most important transcription factor that regulates gene expression under hypoxic conditions. To investigate its role in the glioma cell lines LN229 and U118, we exposed the cells to prolonged hypoxic conditions for 48 h and analyzed the expression of several hub genes using western blotting. Our results, shown in **Figure 8**, demonstrate that the expression of *HIF-1*, *PLAUR*, *SOCS3* and *LIF* was significantly upregulated under prolonged hypoxic conditions. The above evidence proves that hub genes can be modulated under low levels of oxygen by HIF-1.

## Discussion

Tumor hypoxia, characterized by an insufficient supply of oxygen to solid tumors, plays a pivotal role in the development and progression of cancer. This is due to an imbalance between the oxygen demand and supply caused by the rapid growth of tumors. Hypoxia triggers diverse biological processes, including metabolic alterations, angiogenesis, and metastasis, thereby contributing to treatment resistance and unfavorable patient outcomes (34–36). Notably, extensive research has highlighted the profound effect of hypoxia on cancer cells and its potential as an attractive therapeutic target. Moreover, several hypoxia gene signatures have been developed to predict the prognosis of various cancer types, including head and neck, breast, prostate, and bladder cancers. Nonetheless, prior studies have encountered limitations, such as oversimplification of hypoxia gene networks and insufficient integration with conventional prognostic systems. Nevertheless, the significance of tumor hypoxia in prognosis and its potential as a therapeutic target continues to be actively investigated, with substantial promise for enhancing cancer treatment outcomes.

This study comprehensively explored the heterogeneity and molecular characteristics of hypoxic clusters in gliomas. Consensus clustering analysis revealed two distinct clusters, C1 and C2, with C1 being associated with a more aggressive phenotype and poorer prognosis. Differential expression analysis identified genes linked to tumor malignancy, angiogenesis, and epithelial–mesenchymal transition that were upregulated in C1. Immune infiltration analysis showed differences in immune cell composition, indicating higher immunosuppression in C1. Genomic analysis revealed distinct mutation patterns, with C2 exhibiting a higher frequency of *IDH1* and *ATRX* mutations. The prognostic model constructed using LASSO regression and nomogram integration yielded accurate survival predictions. Energy metabolism analysis revealed correlations between hypoxia and glycolysis/oxidative phosphorylation markers. WGCNA identified the blue module as being highly correlated with hypoxia and enriched in wound healing, angiogenesis regulation, and histone modification processes. Hub genes within the blue module (*CLCF1*, *PLAUR*, *SOCS3*, and *LIF*) were found to be modulated by HIF-1 under hypoxic conditions. Overall, this assay enhances our understanding of hypoxic cluster heterogeneity and the molecular characteristics of gliomas. These findings may have implications for targeted therapies and for improving patient outcomes.

Our research focused on investigating the involvement of hub genes in tumor hypoxia, particularly in gliomas. Although these hub genes have been extensively studied in various cancers, their roles in tumor hypoxia remain largely unexplored. For instance, CLCF1 expression has been identified as an independent prognostic factor and a potential target for immunotherapy in hepatocellular carcinoma (37). Additionally, PLAUR promoted anoikis resistance and metastasis in cholangiocarcinoma (34). LIF, a pleiotropic cytokine with diverse roles in different systems, has been implicated in hematopoietic differentiation and has recently emerged as a biomarker and therapeutic target in pancreatic ductal adenocarcinoma (38–40). However, it is important to note that the specific biological functions of these hub genes in the context of tumor hypoxia still require further investigation. Further studies are needed to elucidate the precise roles and mechanisms of tumor hypoxia, potentially opening new avenues for therapeutic interventions and biomarker development.

## Conclusion

In summary, our study introduces a novel hypoxia classification for gliomas, encompassing two well-defined clusters exhibiting distinct prognoses, somatic variations, immune infiltration, and metabolic phenotypes. Our model identified three hub genes that were significantly upregulated under hypoxic conditions. Additional *in vitro* and *in vivo* experiments are required to gain a comprehensive understanding of the underlying molecular mechanisms driving these findings. These studies will provide valuable insights and shed light on the intricate pathways and processes involved in the observed phenomena.

## Data Availability

The original contributions presented in the study are included in the article/**Supplementary Material**. Further inquiries can be directed to the corresponding author.
