# Supplementary material for: Characterization of hypoxia-related molecular clusters and prognostic riskScore for glioma
Source: Front Oncol. 2025 Sep 22;15:1605949. doi: 10.3389/fonc.2025.1605949 (PMC12497635; doi:10.3389/fonc.2025.1605949)
Supplement: Supplementary file 1 [file Table1.docx]

Supplementary Table 1: Univariate cox and Scholfield test for 86 candidates

|  | TCGA cohort | | | | | TCGA cohort | | | | |
| --- | --- | --- | --- | --- | --- | --- | --- | --- | --- | --- |
| Gene | HR | Lower.95CI | Up.95CI | P-value | P-value for Residual analysis | HR | Lower.95CI | Up.95CI | P-value | P-value for Residual analysis |
| ACAT1 | 0.3752 | 0.2763 | 0.5096 | 0 | 0.0943 | 0.465 | 0.3499 | 0.6179 | 0 | 0.1351 |
| ADORA2B | 1.8493 | 1.5864 | 2.1559 | 0 | 0.4373 | 1.5888 | 1.3043 | 1.9354 | 0 | 0.0909 |
| ALDH1A1 | 0.8902 | 0.8041 | 0.9855 | 0.0251 | 0.4296 | 0.7906 | 0.7221 | 0.8656 | 0 | 0.8516 |
| ALDH1A3 | 1.4137 | 1.2911 | 1.548 | 0 | 0.2769 | 1.2677 | 1.1571 | 1.389 | 0 | 0.5358 |
| ANGPTL4 | 1.5339 | 1.4124 | 1.6659 | 0 | 0.1822 | 1.2994 | 1.2017 | 1.405 | 0 | 0.3773 |
| ANXA5 | 2.5147 | 2.226 | 2.8408 | 0 | 0.2637 | 1.9998 | 1.7269 | 2.3159 | 0 | 0.4152 |
| BCL2L1 | 2.2899 | 1.7414 | 3.0113 | 0 | 0.9457 | 2.1643 | 1.6284 | 2.8767 | 0 | 0.1266 |
| BCL2L2 | 0.4703 | 0.4067 | 0.5437 | 0 | 0.0659 | 0.4734 | 0.3843 | 0.5833 | 0 | 0.8693 |
| BIK | 1.6693 | 1.2889 | 2.1621 | 1.00E-04 | 0.6235 | 1.735 | 1.2127 | 2.4823 | 0.0026 | 0.7184 |
| BIRC2 | 2.5391 | 1.8705 | 3.4468 | 0 | 0.4747 | 2.8057 | 2.1248 | 3.7048 | 0 | 0.9485 |
| BNIP3L | 1.4973 | 1.1172 | 2.0066 | 0.0069 | 0.2715 | 1.458 | 1.0966 | 1.9385 | 0.0095 | 0.9308 |
| BPI | 0.3464 | 0.1889 | 0.6354 | 6.00E-04 | 0.2783 | 0.706 | 0.5575 | 0.894 | 0.0039 | 0.3962 |
| BTG1 | 2.1216 | 1.7123 | 2.6288 | 0 | 0.1408 | 1.8762 | 1.5198 | 2.3162 | 0 | 0.6916 |
| CA9 | 1.4589 | 1.379 | 1.5434 | 0 | 0.3904 | 1.2866 | 1.2059 | 1.3727 | 0 | 0.1886 |
| CCNG2 | 0.779 | 0.6299 | 0.9635 | 0.0213 | 0.1504 | 0.7359 | 0.6014 | 0.9006 | 0.0029 | 0.196 |
| CCT6A | 1.6011 | 1.4612 | 1.7543 | 0 | 0.094 | 1.6774 | 1.4537 | 1.9357 | 0 | 0.2956 |
| CITED2 | 2.0551 | 1.7681 | 2.3887 | 0 | 0.1141 | 1.5562 | 1.341 | 1.806 | 0 | 0.9531 |
| COL5A3 | 1.5445 | 1.382 | 1.7261 | 0 | 0.0596 | 1.3643 | 1.2301 | 1.5133 | 0 | 0.443 |
| CTSD | 2.5289 | 2.1074 | 3.0346 | 0 | 0.2322 | 1.7319 | 1.503 | 1.9957 | 0 | 0.9608 |
| DDIT3 | 1.6098 | 1.4592 | 1.776 | 0 | 0.5954 | 1.4954 | 1.3164 | 1.6987 | 0 | 0.6851 |
| DDIT4 | 0.7284 | 0.6489 | 0.8176 | 0 | 0.0831 | 0.8647 | 0.7687 | 0.9726 | 0.0154 | 0.2635 |
| DKC1 | 2.2316 | 1.7525 | 2.8417 | 0 | 0.1221 | 2.8168 | 2.1656 | 3.6639 | 0 | 0.9386 |
| DR1 | 3.789 | 2.8919 | 4.9646 | 0 | 0.3557 | 2.71 | 2.1127 | 3.4762 | 0 | 0.2706 |
| EFNA1 | 1.8399 | 1.5817 | 2.1402 | 0 | 0.7665 | 1.9584 | 1.6601 | 2.3103 | 0 | 0.2248 |
| EGF | 1.8549 | 1.5606 | 2.2046 | 0 | 0.2683 | 1.74 | 1.4505 | 2.0872 | 0 | 0.833 |
| ELF3 | 1.933 | 1.5902 | 2.3498 | 0 | 0.5414 | 1.217 | 1.0491 | 1.4119 | 0.0095 | 0.3606 |
| ELL2 | 1.1942 | 1.0001 | 1.4261 | 0.0499 | 0.3941 | 1.2827 | 1.0912 | 1.5078 | 0.0025 | 0.3174 |
| ENO3 | 1.3824 | 1.154 | 1.656 | 4.00E-04 | 0.8361 | 1.3365 | 1.119 | 1.5963 | 0.0014 | 0.3051 |
| EPO | 2.5241 | 2.113 | 3.0153 | 0 | 0.0686 | 1.5825 | 1.2143 | 2.0623 | 7.00E-04 | 0.2253 |
| FABP5 | 1.6506 | 1.5538 | 1.7533 | 0 | 0.1034 | 1.2423 | 1.1672 | 1.3223 | 0 | 0.3713 |
| FOS | 1.1952 | 1.1039 | 1.2941 | 0 | 0.0658 | 1.1974 | 1.0997 | 1.3037 | 0 | 0.2269 |
| GBE1 | 2.4765 | 2.1157 | 2.8988 | 0 | 0.4427 | 1.8973 | 1.6709 | 2.1543 | 0 | 0.6813 |
| GLRX | 2.5054 | 2.2053 | 2.8465 | 0 | 0.475 | 1.8144 | 1.5585 | 2.1124 | 0 | 0.3977 |
| HERPUD1 | 1.8348 | 1.3966 | 2.4106 | 0 | 0.8674 | 1.3901 | 1.0903 | 1.7723 | 0.0079 | 0.5306 |
| HGF | 1.6501 | 1.4892 | 1.8284 | 0 | 0.1174 | 1.5189 | 1.3669 | 1.6877 | 0 | 0.6891 |
| HIF1A | 1.2401 | 1.0581 | 1.4535 | 0.0079 | 0.777 | 1.2093 | 1.0245 | 1.4274 | 0.0247 | 0.0546 |
| HILPDA | 1.7172 | 1.5825 | 1.8633 | 0 | 0.2049 | 1.3706 | 1.2463 | 1.5073 | 0 | 0.9928 |
| HK2 | 1.7777 | 1.5806 | 1.9993 | 0 | 0.0722 | 1.743 | 1.5548 | 1.954 | 0 | 0.8978 |
| HMOX1 | 1.8191 | 1.6531 | 2.0018 | 0 | 0.0677 | 1.3489 | 1.257 | 1.4476 | 0 | 0.84 |
| HSPD1 | 1.6637 | 1.2017 | 2.3034 | 0.0022 | 0.474 | 1.4884 | 1.0993 | 2.0153 | 0.0101 | 0.1483 |
| IFI27 | 1.5295 | 1.372 | 1.7052 | 0 | 0.0882 | 1.2471 | 1.0928 | 1.4232 | 0.0011 | 0.8333 |
| IGFBP3 | 1.5411 | 1.4378 | 1.6519 | 0 | 0.262 | 1.295 | 1.2199 | 1.3747 | 0 | 0.9381 |
| INSIG1 | 0.7461 | 0.6384 | 0.8719 | 2.00E-04 | 0.4911 | 0.6076 | 0.5175 | 0.7135 | 0 | 0.1024 |
| KDR | 1.3059 | 1.1261 | 1.5145 | 4.00E-04 | 0.1788 | 1.4706 | 1.2437 | 1.7388 | 0 | 0.6136 |
| LOX | 1.6001 | 1.508 | 1.6978 | 0 | 0.2129 | 1.3364 | 1.2571 | 1.4207 | 0 | 0.9793 |
| MPI | 0.4428 | 0.3006 | 0.6521 | 0 | 0.6831 | 0.4431 | 0.3099 | 0.6336 | 0 | 0.0756 |
| MT-CO1 | 0.476 | 0.4104 | 0.5521 | 0 | 0.8214 | 0.3995 | 0.3197 | 0.4992 | 0 | 0.38 |
| MT-CO2 | 0.4383 | 0.3749 | 0.5124 | 0 | 0.6338 | 0.5106 | 0.4239 | 0.6152 | 0 | 0.8935 |
| NDRG1 | 1.3347 | 1.1816 | 1.5076 | 0 | 0.1724 | 1.1467 | 1.0344 | 1.2712 | 0.0092 | 0.1477 |
| NFIL3 | 2.2371 | 1.9171 | 2.6105 | 0 | 0.5017 | 1.9022 | 1.6229 | 2.2297 | 0 | 0.8191 |
| NFKB2 | 1.6349 | 1.3516 | 1.9776 | 0 | 0.6876 | 2.1729 | 1.8396 | 2.5667 | 0 | 0.5293 |
| NOS1 | 0.6517 | 0.5492 | 0.7735 | 0 | 0.6928 | 0.589 | 0.4646 | 0.7466 | 0 | 0.8455 |
| NOS2P2 | 1.3785 | 1.1099 | 1.7121 | 0.0037 | 0.2607 | 1.7684 | 1.3585 | 2.3022 | 0 | 0.7157 |
| P4HA1 | 1.6364 | 1.3661 | 1.9602 | 0 | 0.077 | 1.835 | 1.557 | 2.1627 | 0 | 0.8234 |
| PDGFB | 1.7847 | 1.5081 | 2.1121 | 0 | 0.137 | 1.4335 | 1.2181 | 1.687 | 0 | 0.5784 |
| PFKL | 3.0364 | 2.2376 | 4.1204 | 0 | 0.752 | 2.4979 | 1.8602 | 3.354 | 0 | 0.3785 |
| PGK1 | 2.4565 | 2.1383 | 2.822 | 0 | 0.1085 | 1.6313 | 1.4047 | 1.8945 | 0 | 0.0905 |
| PIM1 | 1.8707 | 1.6624 | 2.1051 | 0 | 0.0774 | 1.9481 | 1.6905 | 2.2451 | 0 | 0.3274 |
| PLAUR | 2.074 | 1.8932 | 2.2721 | 0 | 0.0879 | 1.4584 | 1.3533 | 1.5717 | 0 | 0.3118 |
| PLIN2 | 2.0034 | 1.8176 | 2.2083 | 0 | 0.3759 | 1.4418 | 1.3173 | 1.578 | 0 | 0.3586 |
| PLOD2 | 2.1428 | 1.8972 | 2.4203 | 0 | 0.1918 | 1.7153 | 1.5367 | 1.9147 | 0 | 0.7385 |
| PNN | 0.5835 | 0.482 | 0.7062 | 0 | 0.0534 | 1.451 | 1.1401 | 1.8468 | 0.0025 | 0.3701 |
| POLM | 2.5598 | 2.016 | 3.2501 | 0 | 0.8769 | 1.754 | 1.3514 | 2.2766 | 0 | 0.3453 |
| PPAT | 1.6694 | 1.4344 | 1.9429 | 0 | 0.1595 | 1.5162 | 1.2698 | 1.8105 | 0 | 0.9127 |
| PROK1 | 1.4366 | 1.263 | 1.6341 | 0 | 0.395 | 1.3879 | 1.0995 | 1.7519 | 0.0058 | 0.704 |
| PSMD9 | 5.1777 | 3.7109 | 7.2244 | 0 | 0.073 | 4.3043 | 3.2341 | 5.7287 | 0 | 0.5492 |
| RELA | 1.4431 | 1.0614 | 1.9622 | 0.0193 | 0.6839 | 1.7661 | 1.3402 | 2.3275 | 1.00E-04 | 0.7817 |
| RIOK3 | 4.991 | 3.8137 | 6.5318 | 0 | 0.8657 | 2.6202 | 1.9236 | 3.569 | 0 | 0.9241 |
| RPL36A | 1.3568 | 1.1122 | 1.6553 | 0.0026 | 0.2418 | 1.5167 | 1.1954 | 1.9243 | 6.00E-04 | 0.2153 |
| SAT1 | 2.3467 | 2.0663 | 2.6651 | 0 | 0.1757 | 1.8729 | 1.6504 | 2.1254 | 0 | 0.6586 |
| SGSM2 | 0.4651 | 0.3861 | 0.5602 | 0 | 0.1632 | 0.4486 | 0.3476 | 0.5788 | 0 | 0.0726 |
| SIAH2 | 4.1339 | 3.1831 | 5.3688 | 0 | 0.2521 | 2.2228 | 1.7646 | 2.7999 | 0 | 0.3109 |
| SIRPA | 0.7293 | 0.5966 | 0.8916 | 0.0021 | 0.1788 | 0.5269 | 0.4316 | 0.6432 | 0 | 0.7422 |
| SLC16A1 | 2.2252 | 1.8724 | 2.6444 | 0 | 0.201 | 2.084 | 1.7566 | 2.4724 | 0 | 0.0926 |
| SLC20A1 | 2.5361 | 2.1949 | 2.9304 | 0 | 0.165 | 1.9115 | 1.6799 | 2.1751 | 0 | 0.9049 |
| SLC3A2 | 2.0552 | 1.5867 | 2.6621 | 0 | 0.6342 | 1.3796 | 1.0765 | 1.768 | 0.011 | 0.3405 |
| SYT7 | 0.9148 | 0.8442 | 0.9914 | 0.03 | 0.7094 | 0.7598 | 0.6921 | 0.8342 | 0 | 0.6328 |
| TBPL1 | 0.3304 | 0.2602 | 0.4195 | 0 | 0.7107 | 0.6336 | 0.5109 | 0.7857 | 0 | 0.8426 |
| TCEAL1 | 0.4254 | 0.3065 | 0.5906 | 0 | 0.333 | 0.4765 | 0.3646 | 0.6228 | 0 | 0.2587 |
| TF | 0.7957 | 0.7384 | 0.8575 | 0 | 0.7771 | 0.8493 | 0.7929 | 0.9098 | 0 | 0.3132 |
| TFF3 | 1.3061 | 1.1889 | 1.435 | 0 | 0.2192 | 1.2267 | 1.0555 | 1.4257 | 0.0077 | 0.2529 |
| TFRC | 2.4075 | 2.1207 | 2.7331 | 0 | 0.7641 | 1.4666 | 1.2375 | 1.738 | 0 | 0.5197 |
| TGFA | 0.8408 | 0.7356 | 0.9611 | 0.011 | 0.3434 | 0.6821 | 0.5879 | 0.7914 | 0 | 0.272 |
| TP53 | 1.6943 | 1.4422 | 1.9905 | 0 | 0.0775 | 1.3977 | 1.2018 | 1.6255 | 0 | 0.8906 |
| VEGFA | 1.5727 | 1.4779 | 1.6735 | 0 | 0.3367 | 1.2917 | 1.2176 | 1.3703 | 0 | 0.6265 |
| VEGFC | 1.3817 | 1.17 | 1.6317 | 1.00E-04 | 0.2498 | 1.5507 | 1.2608 | 1.9072 | 0 | 0.6638 |

Supplementary Table 1 Clinical and molecular characteristics of patients included in this study

| Dataset | TCGA RNA-Seq | CGGA RNA-Seq | GTEx RNA-Seq |
| --- | --- | --- | --- |
|  |  |  |  |
| **Formation** |  |  |  |
| Glioma tissue | 697 | 325 | 0 |
| Non-tumor tissue | 5 | 20 | 200 |
|  |  |  |  |
| **Gender** |  |  |  |
| Male | 288 | 122 |  |
| Female | 394 | 203 |  |
| Unavailable | 15 | 0 |  |
|  |  |  |  |
| **Age(years)** |  |  |  |
| Means | 60 | 43 |  |
| Range | 14-89 | 8-81 |  |
|  |  |  |  |
| **Survival status** |  |  |  |
| Alive | 414 | 99 |  |
| Dead | 235 | 219 |  |
| Unavailable | 48 | 7 |  |
|  |  |  |  |
| **Overall survival(month)** |  |  |  |
| Median | 48,6 | 37.37 |  |
|  |  |  |  |
| **Histology** |  |  |  |
| Astrocytoma | 254 | 62 |  |
| Oligodendroglioma | 258 | 52 |  |
| GBM | 151 | 139 |  |
| Unavailable | 45 | 4 |  |
|  |  |  |  |
| WHO grade |  |  |  |
| II | 254 | 109 |  |
| III | 262 | 72 |  |
| IV | 153 | 144 |  |
| Unavailable | 28 | 0 |  |
|  |  |  |  |
| **IDH-1 mutation & 1p/19q codeletion status** |  |  |  |
| IDHmut-non-codel | 255 | 109 |  |
| IDHmut-codel | 169 | 63 |  |
| IDHwt | 229 | 145 |  |
| Unavailable | 44 | 8 |  |
|  |  |  |  |
|  |  |  |  |
|  |  |  |  |
|  |  |  |  |
|  |  |  |  |
|  |  |  |  |
|  |  |  |  |

Supplementary Table 3 Reactome enrichment analysis results of the top 20 mutated genes in C1 patients.
